# AVPCD: a plant-derived medicine database of antiviral phytochemicals for cancer, Covid-19, malaria and HIV

**DOI:** 10.1093/database/baad056

**Published:** 2023-08-18

**Authors:** Shahid Ullah, Wajeeha Rahman, Farhan Ullah, Anees Ullah, Gulzar Ahmad, Muhammad Ijaz, Hameed Ullah, Zilong Zheng, Tianshun Gao

**Affiliations:** S Khan Lab Mardan, Khyber Pakhtunkhwa, Takhtbhai, KP 23200, Pakistan; S Khan Lab Mardan, Khyber Pakhtunkhwa, Takhtbhai, KP 23200, Pakistan; S Khan Lab Mardan, Khyber Pakhtunkhwa, Takhtbhai, KP 23200, Pakistan; S Khan Lab Mardan, Khyber Pakhtunkhwa, Takhtbhai, KP 23200, Pakistan; S Khan Lab Mardan, Khyber Pakhtunkhwa, Takhtbhai, KP 23200, Pakistan; S Khan Lab Mardan, Khyber Pakhtunkhwa, Takhtbhai, KP 23200, Pakistan; S Khan Lab Mardan, Khyber Pakhtunkhwa, Takhtbhai, KP 23200, Pakistan; Big Data Center, The Seventh Affiliated Hospital of Sun Yat-sen University, Shenzhen 518107, P. R. China; Big Data Center, The Seventh Affiliated Hospital of Sun Yat-sen University, Shenzhen 518107, P. R. China

## Abstract

Serious illnesses caused by viruses are becoming the world’s most critical public health issues and lead millions of deaths each year in the world. Thousands of studies confirmed that the plant-derived medicines could play positive therapeutic effects on the patients with viral diseases. Since thousands of antiviral phytochemicals have been identified as lifesaving drugs in medical research, a comprehensive database is highly desirable to integrate the medicinal plants with their different medicinal properties. Therefore, we provided a friendly antiviral phytochemical database AVPCD covering 2537 antiviral phytochemicals from 383 medicinal compounds and 319 different families with annotation of their scientific, family and common names, along with the parts used, disease information, active compounds, links of relevant articles for COVID-19, cancer, HIV and malaria. Furthermore, each compound in AVPCD was annotated with its 2D and 3D structure, molecular formula, molecular weight, isomeric SMILES, InChI, InChI Key and IUPAC name and 21 other properties. Each compound was annotated with more than 20 properties. Specifically, a scoring method was designed to measure the confidence of each phytochemical for the viral diseases. In addition, we constructed a user-friendly platform with several powerful modules for searching and browsing the details of all phytochemicals. We believe this database will facilitate global researchers, drug developers and health practitioners in obtaining useful information against viral diseases.

## Introduction

**Figure 1.**
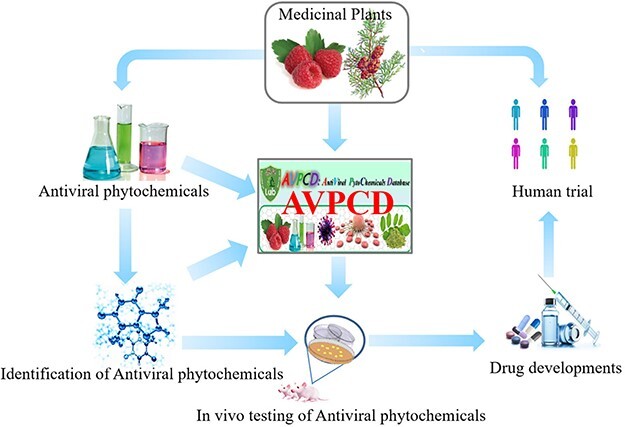
The graphical abstract of the AVPCD.

Nature is a unique origin of systems with a wide range of phytochemical diversity, many of which have fascinating biological activities and medicinal properties. Phytochemicals are bioactive, naturally occurring chemical compounds, which are present in plants and provide health benefits to humans (1). It is present in different parts of the plants, such as in the roots, stems, leaves, rhizome, flowers, fruits or seeds. The plant kingdom is a great source of potential drugs, and there has been a significant role of medicinal plants in recent years. Plant-based drugs are widely available, less expensive, safe and efficient with few side effects (2, 3). Phytochemicals benefit plants by performing secondary functions, including assisting in plant growth, protecting plants by activating defense mechanisms and imparting color, odor and flavor to the plants (4). According to traditional medicinal practices as well as modern scientific studies, they are useful for medicinal purposes to alleviate diseases and improve human health. These plants are thought to be rich sources of phytochemicals that can be used in the synthesis and production of drugs (5, 6). These phytochemicals, such as flavonoid, quinine, quercetin and terpenoid, perform good biological functions with many therapeutic activities, including anti-cancer, anti-HIV, anti-malaria and anti-Covid (7). Each year, tens of millions of people are infected with different viruses including COVID, malaria, HIV, cancer-inducing viruses, which cause 571 million, 241 million, 37.9 million and 18.1 million infections, respectively. Commonly used antivirals often have limited efficacy and serious side effects, while herbal extracts have been used for medicinal purposes since ancient times and are known for their antiviral properties and tolerable side effects (8, 9). The spread of viral diseases is a worldwide concern, requiring a critical need for the most promising antivirals. Some viral diseases can be cured with approved antiviral drugs, but others have no vaccines or drugs available. The majority of approved antiviral drugs are associated with side effects, which eventually raise the need for the development of antiviral based on natural phytochemicals (10, 11). Most viral diseases, such as HIV, cancer, malaria and Covid-19, as well as other diseases caused by alphaviruses, flaviviruses and plasmodium, are posing a significant risk. Coronavirus disease (COVID-19), caused by a newly identified coronavirus, recently became pandemic and severely impacted the world’s population. Due to viruses’ ability to mutate their genomes and become resistant to drugs, developing effective treatments and antivirals against viruses has become difficult in the recent years (12–14). Antiviral drugs also have side effects that affect human health both directly and indirectly. This leads toward the development of plant-based drugs and herbal treatments with few side effects (15, 16). Computational resources are a valuable source of data, expertise and information in biological research, especially for medicinal plants due to their different medicinal properties (17). Several databases have been published in this field of research such as MAPS (18), MPD3 (19), IMPPAT (20) and TIPdb (21) that have much more phytochemical data. However, we have presented a special platform for antiviral phytochemical and have collected almost all the data from 1980 to 2021. Previously, we have also published numerous biological databases of different research area, which are Co-19pdb, dbpaf, CGDB, DBPSP and so on. In this work, we have tried to provide a friendly platform for global researchers, drug developers, health practitioners and students with very frequent updates to add in their study and research, which is a comprehensive collection of 2537 antiviral phytochemicals from 383 medicinal plants and 319 multiple families, including their scientific, family and common names, as well as their utilized parts, disease information, PubMed IDs or links of relevant articles, compound summary including 2D & 3D structure, molecular formula, molecular weight, isomeric SMILES, InChi, Inchikey, IUPIC name and 21 other properties. We have used different keywords such as ‘Medicinal plant’, ‘phytochemical’, ‘plant-derived compound’, ‘COVID related drugs plant’, ‘cancer related medicinal plant’, ‘malaria medicinal plant’ in several search engines including Google, Google Scholar, PubMed, Science Direct and Bing for searching. Finally, we have provided a comprehensive database, which is built in JavaScript, PHP, HTML and CSS, which will be updated timely. The graphical image Figure 1 is the stepwise representation of the medicinal plants to final drugs and human trial, while Table 1 shows all the statistics of the data.

**Table 1.** The number of collected data

AVPCD	Anti-cancer	Anti-malarial	Anti-HIV	Anti-COVID	Others	Total
Phytochemicals	319	586	1478	109	45	2537
Medicinal plants	97	180	84	13	9	383
Family	97	116	84	13	9	319

## Result and discussion

### Construction of AVPCD

**Figure 2.**
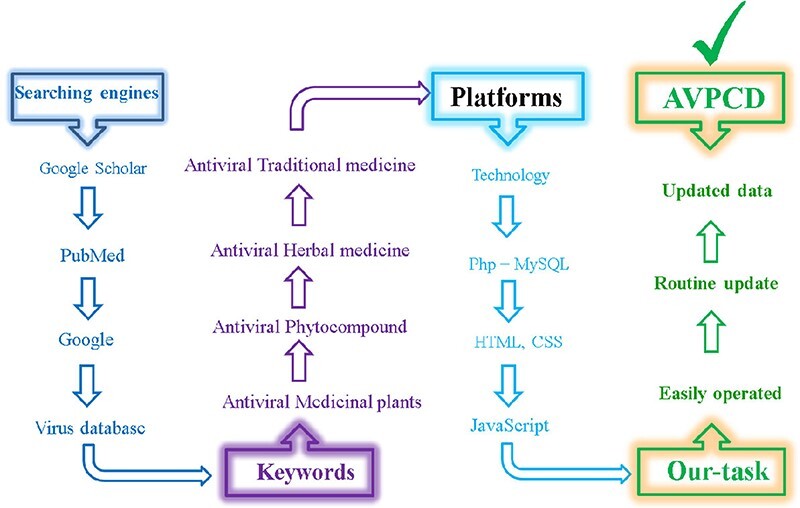
Color-wise and graphical representation of the collection of antiviral phyto-compounds and construction of AVPCD.

We integrated the data from multiple sources, including PubMed, Google, Google Scholar and so on. We have used various keywords such as ‘Antiviral Medicinal plants’, ‘Antiviral Phytocompound’, ‘Antiviral Herbal medicine’, ‘Antiviral Traditional medicine’ and ‘databases of Antiviral Phytocompound’ to search and retrieve published antiviral-phytocompound-related data with the help of literature database of PubMed (http://www.ncbi.nlm.nih.gov/pubmed). To circumvent missing data, we have manually collected the latest data from The New Phycologist (https://nph.onlinelibrary.wiley.com/), Bioresource Technology (https://www.elsevier.com/journals/bioresource-technology), Nucleic Acids Research (NAR) (https://academic.oup.com/nar) and journal of Genomics, Proteomics & Bioinformatics (GPB) (https://www.journals.elsevier.com/genomics-proteomics-and-bioinformatics), which are the leading research journals on database issue. To obtain antiviral phytochemicals of high quality, we collected only the experimentally validated compounds and removed the ones with broken links. Multiple programming languages including PhP, MYSQL, HTML, CSS and JavaScript have been used to construct the database. Figure 2 depicts all of the steps for collecting the data and creating the database, both in terms of colors and aesthetics. Finally, we supplied to the scientific community a compressive antiviral phytochemical research database that is simple to use and will be updated over time.

## Usage of the AVPCD database

### Search option of AVPCD

**Figure 3.**
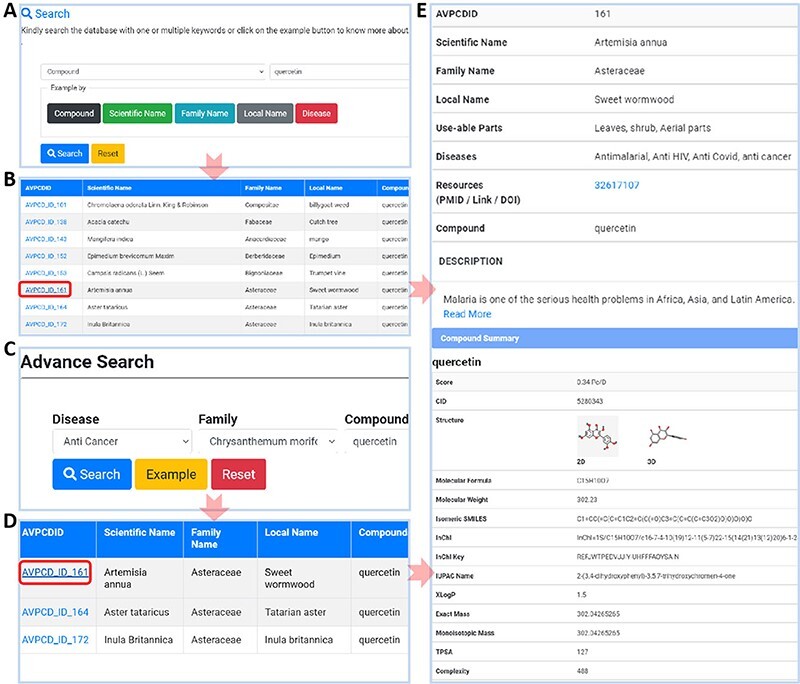
Usage of AVPCD. (A) The simple search with five search options. (B) The compound list by the selected option in simple search. (C) The advanced search of the AVPCD. (D) The compound list by the advanced search. (E) All the needed and final data of the searched query.

AVPCD is designed in an easy and user-friendly way for searching and browsing the data. To know more in depth, users could search five classic examples including ‘quercetin’, ‘Sedum sarmentosum’, ‘Aseraceae’, ‘Lobelia’ and ‘Anti HIV’ by clicking ‘compound’, ‘scientific name’, ‘Family name’, ‘Local Name’ and ‘Disease’ buttons, as shown in Figure 3A, respectively. Users could get the result by selecting the compound of interest in Figure 3B. Further clicking the highlighted AVPCD ID will bring the user to the final result, shown in Figure 3E. For special search, we provided the ‘Advance Search’ option, shown in Figure 3C, with fixed ‘Anti-Cancer’ as an example, and on further click it will open a new window with the compound list, as in Figure 3D. Clicking the compound of interest will link to a new page with all required knowledge of this compound, such as scientific, local and common name from obtained plants, utilized part, disease for used, original resources with short introduction of the needed plants, compound summary, compound score, 2D and 3D structure, molecular formula, molecular weight, isomeric SMILES, InChI, InChI Key, IUPIC name and 21 other properties, shown in Figure 3E.

### Browse option of AVPCD

**Figure 4.**
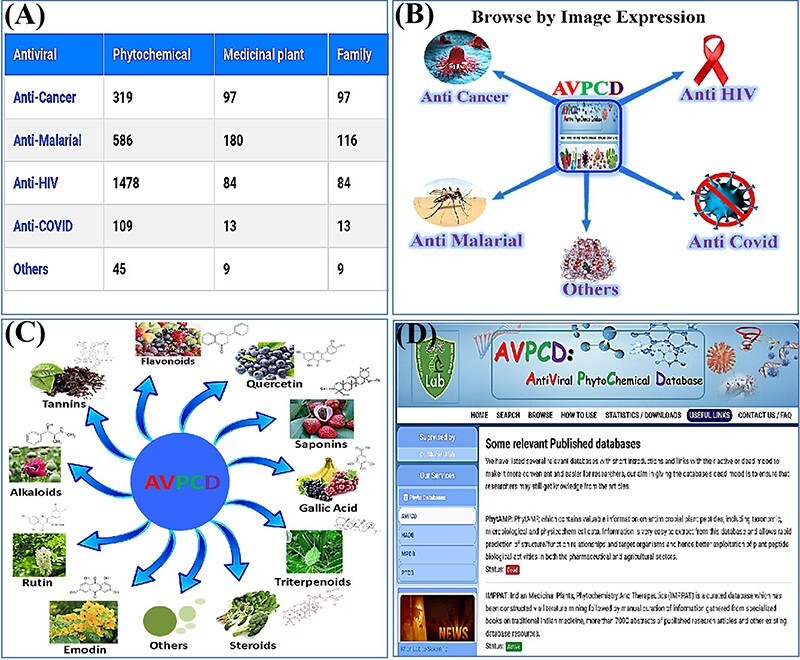
Usage of the database. (A) The browse option by antiviral category. (B) Browse by disease-wise image expression. (C) Browse page of the AVPCD based on the top 10 rich compound images and formula. (D) Useful links to the relevant databases with two modes.

Three different browse options are available to browse the AVPCD data. To obtain the whole result of each antiviral, users can click on the browse option and then directly get all the needed information by clicking any antiviral shown in Figure 4A. Furthermore, we gave the image expression browse option shown in Figure 4C, and clicking the required category will lead to the new window and final result. We also fixed the browse option by top 10 rich compound image with formula shown in Figure 4C. To make it easier and more authentic, we provided several options on the main bar of the database, including the ‘Usage’ showing the detail with visual image expression of database, ‘statistics’ displaying the statistics of the database, ‘download’ giving all the data for scientific research only after publishing the article and ‘useful links’ presenting the published databases of this research area with two buttons. Clicking the active button will give the database while the dead button will bring users to the broken database article (Figure 4D).

### The biological activities

**Figure 5.**
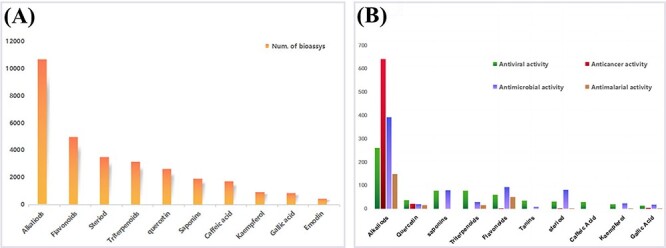
The top 10 compounds with different biological activities. (A) The bioassays of top 10 compounds. (B) The bioassays of mentioned antiviral compounds in AVPCD.

We analyzed the bioassays of top 10 compounds, including Alkaliods, Quercetin, Saponins, Flavonoids, Tanins, Triterpenoids, Steriod, Caffeic Acid, Kaempferol and Gallic Acid. We checked the total biological activities as well as specific mentioned antiviral effects, among them. Alkaliods, Flavonoids, Steriods and Triterpenoids are the more active compounds that have more correlation with diseases (Figure 5A). Moreover, we have specified the antiviral activity, anticancer, antimicrobial, antimalarial activity of the top 10 compounds, in which Alkaliods are the most useful compounds for all diseases and highly active for anticancer antimicrobial, antiviral and antimalarial. Quercetin is the second active compound that can be used in anticancer antimicrobial, antiviral and antimalarial. The details of all top compounds are shown in Figure 5B.

### Statistics of the AVPCD

**Figure 6.**
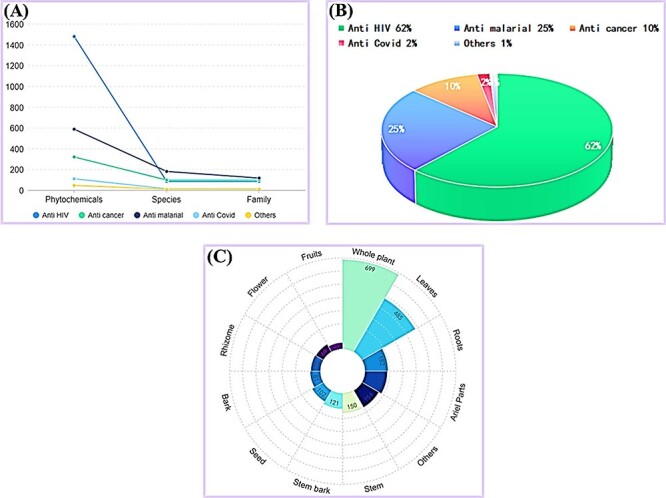
The statistics of AVPCD, (A) The number of antiviral phytochemicals, species and family. (C) The percentage of antiviral phytochemical. (D) The number of all usable parts of medicinal plants.

In the current work, we have a comprehensive collection of total 2537 antiviral phytochemicals from a total of 383 medicinal plants and total 319 multiple families, including their scientific, family and common names, as well as their utilized parts, disease information, active compounds, PubMed IDs or links of the relevant articles. We also compared the numbers of phytochemical, species and family among the five classifications, in which ‘anti HIV’ is on the top (Figure 6A). Figure 6B shows the percentage of all antiviral phytochemical, while Figure 6C presents degree of use for different usable parts of the medicinal plants.

### Aims of the AVPCD database

The AVPCD intended to collect information in a uniform manner regarding all published experimental antiviral phytochemicals, including plant species, their scientific local and common names and areas, especially the bioassays of antiviral phytochemical compounds. This database includes information on the species taxonomy, distribution, ecology, collection records, analytical data and references to previous studies and will be updated on a regular basis with new findings and more information. Further, we have focused on creativity, consistency and clarity for which we are working on voice interface designing, regional language search option, as well as API’s, as we previously published multiple datasets on various research areas in highly referenced international journals, including a database of circadian genes in eukaryotes (CGDB) in *Nucleic Acids Research* (22), cancer research database (CRDB) in *JMIR Cancer* (23), databases on phosphorylation animal and fungi (DBPAF) in *Scientific Report* (24), Latest Database of Protein Research (LDBPR) in *Journal of Bioinformatics and Systemic Biology* (25), database for protein phosphorylation sites in prokaryotes (dbPSP), in *Database (Oxford)* (26), COVID-19 Pandemic Database (CO-19PDB) in *Computer Methods Programs and Biomedicine Updates* (27), Database relevant to Human Research (DBHR) in *Future Science OA* (28). In all, we have provided a huge platform of 19 databases of different research area named ‘Home of all Biological Databases’ (HABD) (29, 30) (www.habdsk.org) in Table 2, which gave free access for global scientific community. Therefore, we felt to give comprehensive databases in this field of research as well; for that, AVPCD aims to provide wonderful insights for researchers with well-gathered previously published work in one platform. We tried to provide access to data from sources that are difficult to locate. AVPCD gives details that may not have been published before on such easy and friendly way in the open literature. AVPCD monitored and updated the dead and broken databases and have provided a separate page with two moods. Clicking the active mood will give the database and dead mood will bring the articles with more knowledge, evidence or citations.

**Table 2.** All databases of the HADB site

Db title	Databases links	Databases category	Status
1) Compendious databases			
DBHR	https://www.habdsk.org/dbhr	Human	(28)
LDBPR	https://www.habdsk.org/ldbpr	Protein	(25)
Co-19PDB	https://www.habdsk.org/co-19pdb	Covid-19	(27)
CRDB	https://www.habdsk.org/crdb	Cancer and Covid	(23)
Edbco-19	https://www.habdsk.org/edbco-19	Covid-19	(64)
FDBC	https://www.habdsk.org/fdbc	Fungi	(65)
DBPR	https://www.habdsk.org/db-pr	Plant	Submitted
DBBT	https://www.habdsk.org/dbbt	Biological tools	Ongoing
CO-19PDB 1.0	https://www.co-19pdb.habdsk.org/	Covid-19	Ongoing
CBDB	https://www.habdsk.org/cbdb	Biological DB	Ongoing
DBFBPA	https://www.habdsk.org/db-fbpa	Fungus, Bacteria, Protozoa	Ongoing
BDSR	https://www.habdsk.org/bdsr	Biological Db	Ongoing
CDB-PTMS	https://www.habdsk.org/cdb-ptms	PTMs	Ongoing
CHCRD	https://www.habdsk.org/chcrd	Cancer	Submitted
2) Phyto-databases			
AVPCD	https://avpcd.habdsk.org/	Antiviral	This one
HADB	https://hadb.habdsk.org/	Hyper-accumulators	———
MPDB	https://www.mpdb.habdsk.org/	Medicinal plant	———-
PTDB	https://www.ptdb.habdsk.org/	Phytotoxin	———
3) Others databases			
PBDB	https://pbdb.habdsk.org/	Plastic biodegrading	———-

### The advantages of AVPCD against other databases

Although many useful databases have been published in the phytochemical research area, none of them is specially designed for antivirals. In order to accomplish this, we attempted to develop a comprehensive special platform for antiviral data and to allow easy access to the scientific community with rapid updates. We have provided all antiviral compound information that has not previously been published in such an easy manner. Regarding previous databases, some of them only supplied phyto-compounds, some plants and some parts, and the majority of them had limited antiviral data, so we felt that in this pandemic, we should give a separate and dedicated platform specifically for viral diseases. Our database solely contains information about antiviral diseases and compounds.

### AVPCD phyto-compounds scoring system

Various scoring systems have been presented in published work, for example, molecular docking scoring of some phyto-compounds (31–33), experimental work evidence of the top 10 compounds, on the basis of which we have given the special scoring system and have presented it in AVPCD; we have used the following formula and have cross checked all the data with published work. Artemisinins ranked first in our score, and a Chinese scientist was awarded the Nobel Prize in 2016 for it (34–36), followed by keampferol, used to treat a variety of disorders (37–40), Chlorogenic acids (41–43), Lupeol (44–46), Oleanolic acid (47–49), Robusta flavone, Cucurbitacins (50–53), Rutinosid (54, 55), Morelloflavone (56–58), Betulinic (59–61) and Hinokiflavone (62, 63); Jaccard similarity formula is used to measure the correlation between any two phyto compounds (e.g. ${P_i}$and ${P_j}$) based on the overlap in the same study. The Jaccard similarity between ${P_i}$and ${P_j}$ can be calculated as


$${J_{{p_i}{p_j}}} = \frac{{\left| {{P_i}\mathop \cap \nolimits^ {P_j}} \right|}}{{\left| {{P_i}\mathop \cup \nolimits^ {P_j}} \right|}} = \frac{{\left| {{P_i}\mathop \cap \nolimits^ {P_j}} \right|}}{{\left| {{P_i}} \right| + |{P_j}\left| - \right|{P_i}\mathop \cap \nolimits^ {P_j}|}}$$

where$\left| {{P_i}} \right|$, $\left| {{P_j}} \right|{\ }$and $\left| {{P_i}\mathop \cap \nolimits {P_j}} \right|$ represent the number of the studies in ${P_i}$, ${P_j}$ and their overlap, respectively. For a single Phyto-compound, its Jaccard similarity matrix of combination of all the data was integrated as


$$\left[ {\begin{array}{*{20}{c}}{\begin{array}{*{20}{c}}{{J_{{p_1}{p_1}}}}& \cdots &{{J_{{p_1}{p_i}}}}\\\vdots &{\ }&{{\ } \vdots }\end{array}}&{\begin{array}{*{20}{c}}\cdots &{{J_{{p_1}{p_{n{\rm{ }}}}{\ }}}}\\
{\ }& \vdots \end{array}}\\{\begin{array}{*{20}{c}}{{J_{{p_i}{p_1}}}}& \cdots &{{J_{{p_i}{p_i}}}}\\{{\ } \vdots }&{\ }& \vdots \\{{J_{{p_n}{p_1}}}}& \cdots &{{J_{{p_n}{p_i}}}}\end{array}}&{\begin{array}{*{20}{c}}\cdots &{{\ }{J_{{P_i}{p_n}{\ }}}}\\{\ }& \vdots \\\cdots &{{J_{{p_n}{p_n}}}}
\end{array}}\end{array}} \right]$$

Using the Jaccard matrix, we measured the strength of any Phyto-compound ${P_i}$ as


$${S_{{{\rm{p}}_{\rm{i}}}}} = \frac{{\sum \nolimits_{J = 1}^{n} J_{P_i}{p_j}}}{{\sum \nolimits_{J = 1,k = 1}^{n}{J_{{p_j}{p_k}}}}}{\ }\left( {J,k \in \left[ {1,n} \right],J \ne i,J \ne k} \right)$$

By combination of all phyto-compounds in one final score, the signal score of any phyto-compound ${\rm{i}}$ could be defined as


$${S_{score}}\left( i \right) = \mathop \sum \limits_{j = 1}^n {S_{{p_j}}}{S_{{p_j}}}\left( i \right)$$

where ${S_{{p_j}}}\left( i \right)$ represents the signal score of the disease $i{\ }$in the phyto-compound${p_j}$.

## Future direction

**Figure 7.**
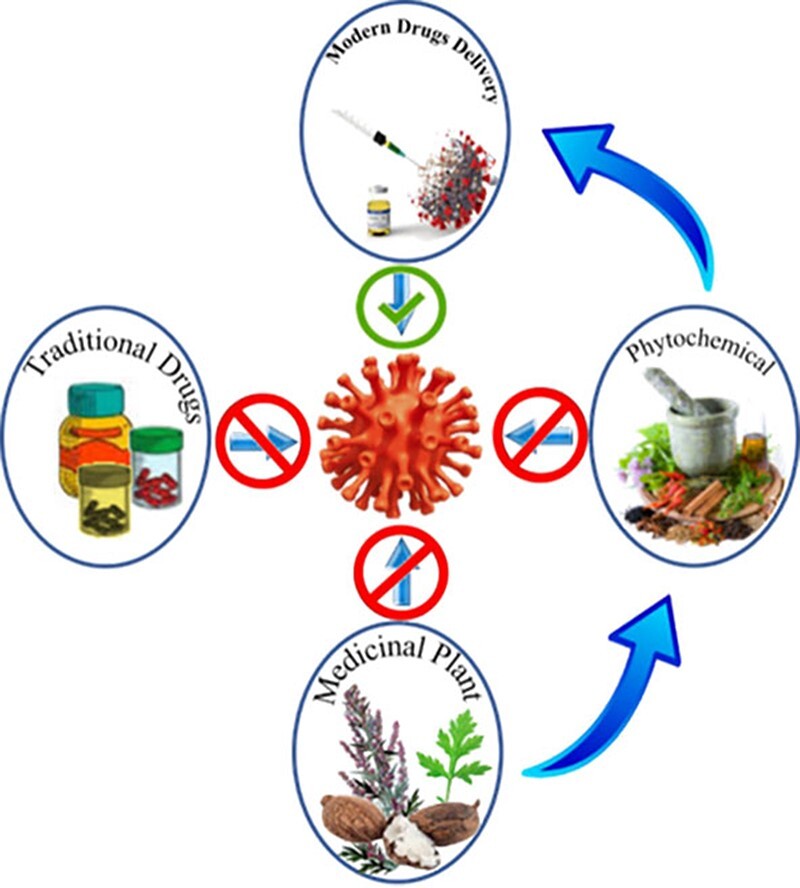
The future direction of the antiviral phytochemicals.

Many traditional medicines and phytochemicals are directly utilized to treat various ailments; however, the majority of them have efficacy and safety concerns. In Figure 7, we depict the future trend for antiviral phytochemicals that can be used and will be more advantageous for modern medications. Interferon and ribavirin, for example, are efficient *in vitro* against most viruses but are frequently unsuccessful in humans. Today’s antiviral medications (3, 4) address only a subset of viruses, including HIV, herpes viruses such as HSV, human cytomegalovirus (hCMV), varicella zoster virus (VZV), influenza viruses and hepatitis viruses. There is currently no approved treatment for many types of viruses, and vaccination is confined to hepatitis A, mumps and varicella. Furthermore, these drugs are frequently expensive and ineffectual due to viral resistance, and they induce side effects. Keeping this in mind, natural-based pharmacotherapy may be a viable option for treating viral infections. As a result, more research into antiviral phytochemicals is required, with a focus on drug delivery applications in overcoming many biological obstacles that exist for antiviral medicines to successfully reach their intended site(s) of action. The current study focuses on the antiviral capabilities of herbal extracts and bioactive ingredient isolates derived from medicinal plants, as well as initiatives to improve their administration.

## Conclusion

A biological database is a collection of tools for storing, organizing and retrieving biological data and other types of information so that it may be conveniently examined, controlled and amended. A number of articles have been published in this field of research, each of which has its own collocation of data based on function, use, technical factors and species. For such works, we have provided a well-managed database that aimed to maintain medicinal plants and other phytochemical-tolerant plants; timely identification is required in order to investigate their unique physiological systems and take benefit of their unique characteristics. Further, our database provides statistical support for the existence of antiviral phytochemical and medicinal plants in global, regional and local floras based on disease concentrations. In addition, we have provided every single compound summary including 2D and 3D structure, molecular formula, molecular weight, isomeric SMILES, InChI, InChI key, IUPAC name and 21 other properties; we have collected almost all the data with their relevant knowledge and have provided a separated page with a short introduction and have updated or removed all broken and unverified data. Furthermore, AVPCD offers two search and three browse options in an easy and friendly finding manner, and will be updated in time, that can be access through this link https://www.avpcd.habdsk.org/.

## Data Availability

These data will be available under the journal rule and regulation.
